# Out-of-range INR values and outcomes among new warfarin patients with non-valvular atrial fibrillation

**DOI:** 10.1007/s11096-014-0038-3

**Published:** 2014-11-27

**Authors:** Winnie W. Nelson, Li Wang, Onur Baser, Chandrasekharrao V. Damaraju, Jeffrey R. Schein

**Affiliations:** 1 Health Economics & Outcomes Research (HECOR), Janssen Scientific Affairs, LLC, 1000 US Highway 202 South, Room 3264, Raritan, NJ 08869 USA; 2 STATinMED Research, Plano, TX USA; 3 STATinMED Research, Ann Arbor, MI USA; 4 MEF University, Istanbul, Turkey; 5 Janssen Research and Development, LLC, Raritan, NJ USA

**Keywords:** Atrial fibrillation, Clinical outcomes, International normalized ratio, USA, US veterans, Warfarin

## Abstract

*Background* Although efficacious in stroke prevention in non-valvular atrial fibrillation, many warfarin patients are sub-optimally managed. *Objective* To evaluate the association of international normalized ratio control and clinical outcomes among new warfarin patients with non-valvular atrial fibrillation. *Setting* Adult non-valvular atrial fibrillation patients (≥18 years) initiating warfarin treatment were selected from the US Veterans Health Administration dataset between 10/2007 and 9/2012. *Method* Valid international normalized ratio values were examined from the warfarin initiation date through the earlier of the first clinical outcome, end of warfarin exposure or death. Each patient contributed multiple in-range and out-of-range time periods. *Main outcome measure* The relative risk ratios of clinical outcomes associated with international normalized ratio control were estimated. *Results* 34,346 patients were included for analysis. During the warfarin exposure period, the incidence of events per 100 person-years was highest when patients had international normalized ratio <2:13.66 for acute coronary syndrome; 10.30 for ischemic stroke; 2.93 for transient ischemic attack; 1.81 for systemic embolism; and 4.55 for major bleeding. Poisson regression confirmed that during periods with international normalized ratio <2, patients were at increased risk of developing acute coronary syndrome (relative risk ratio: 7.9; 95 % confidence interval 6.9–9.1), ischemic stroke (relative risk ratio: 7.6; 95 % confidence interval 6.5–8.9), transient ischemic attack (relative risk ratio: 8.2; 95 % confidence interval 6.1–11.2), systemic embolism (relative risk ratio: 6.3; 95 % confidence interval 4.4–8.9) and major bleeding (relative risk ratio: 2.6; 95 % confidence interval 2.2–3.0). During time periods with international normalized ratio >3, patients had significantly increased risk of major bleeding (relative risk ratio: 1.5; 95 % confidence interval 1.2–2.0). *Conclusion* In a Veterans Health Administration non-valvular atrial fibrillation population, exposure to out-of-range international normalized ratio values was associated with significantly increased risk of adverse clinical outcomes.

## Impacts on practice

- Non-valvular atrial fibrillation patients exposed to a warfarin international normalized ratio of less than two have significantly increased thrombosis risk.
- The adverse event risk was greater for non-valvular atrial fibrillation patients with below-range international normalized ratio than those with above-range international normalized ratio. Awareness of this excess risk may better inform clinicians regarding appropriate therapeutic approach options.

## Introduction

Atrial fibrillation (AF) is the most common heart dysrhythmia in the United States. The American Heart Association estimated that 2.7 million Americans are diagnosed with AF [1]. AF prevalence has increased as the US population has aged, and is expected to continue to rise in the coming decades [2].

Patients with AF have an almost five-fold increase in the risk of stroke when compared to those without AF [3]. Current guidelines recommend long-term anticoagulation treatment for the prevention of stroke among moderate- and high-risk AF patients [4]. Several large-scale clinical trials have shown the effectiveness of warfarin in reducing stroke associated with AF [5–7]. A meta-analysis conducted by Hart et al. [8] in 1999 demonstrated that adjusted-dose warfarin reduced ischemic stroke by 65 % in AF patients. To maximize benefits and minimize complications of prophylaxis, warfarin dosing should be closely monitored and adjusted to maintain the international normalized ratio (INR) [9] between 2.0 and 3.0 for non-valvular (NV) AF patients [10].

## Aim of the study

Few studies have focused on the effect of out-of-range INR on important major adverse cardiac outcomes such as acute coronary syndrome (ACS) and transient ischemic attack (TIA), in addition to stroke and bleeding. This study explored the association of INR control and clinical outcomes among NVAF patients who newly initiated warfarin prophylaxis.

## Ethical approval

No patient identity or medical records were disclosed for the purposes of this study, except in compliance with applicable law. Since the core study proposed herein does not involve the collection, use, or transmittal of individual identifiable data, Institutional Review Board (IRB) approval to conduct this study is not required. Both the data set and the security of our offices where we keep the data set meet the requirements of the Health Insurance Portability and Accountability Act (HIPAA) of 1996.

## Methods

This study report was written in compliance with the STrengthening the Reporting of OBservational studies in Epidemiology (STROBE) statement [11].

### Data source

The Veterans Health Administration (VHA) dataset was used to access medical, pharmacy, laboratory and enrollment information. The VHA is the largest integrated health care system in the United States, providing care for 5 million patients across the country [12]. Electronic health data collected within the VA national Medical SAS^®^ Dataset and Decision Support System were evaluated. These data include hospital and outpatient diagnoses, procedures, laboratory results and dispensed medications. Death date was determined using the VA Vital Status file, which ascertains mortality using the Social Security Death Master File, Medicare Vital Status Files, and VA Beneficiary Identification and Records Locator Subsystem.

### Study population

The study population consisted of patients aged 18 years or older with at least one pharmacy claim for warfarin from October 1, 2008 to September 30 2011. Only incident warfarin users were included for study; prevalent warfarin users were excluded. Study patients were required to have at least one valid INR measurement (0.5 ≤ INR ≤ 20) within 14 days after the initial warfarin pharmacy claim date (index date) and at least three INR measurements. Included patients must have had continuous health care coverage for 12 months prior to the index date, the period that would serve as the baseline period.

The population was further restricted to patients with at least one medical claim for AF [International Classification of Diseases, 9th revision, Clinical Modification (ICD-9-CM) code 427.31] between 30 days before the index date through the end of the follow-up period. Patients with at least one pharmacy claim for warfarin during the baseline period and those diagnosed with transient or perioperative AF disease, mitral or aortic valve repair or replacement, at least two claims for hyperthyroidism, record of pregnancy or delivery at any time during the study period were excluded.

### Study Measures

The warfarin exposure time during the follow-up period was estimated based on an algorithm commonly used in AF studies by Go et al. [13]. Warfarin exposure duration corresponded to the prescription date plus days’ supply and 45 days. INR coverage was assumed to be 45 days after the INR measurement date. If a patient had a gap >45 days between two consecutive warfarin exposure periods, therapy was considered discontinued before the gap. For patients who restarted warfarin therapy after discontinuation, only the first warfarin exposure period was considered.

INR results were observed from the index date through the earlier of the first clinical outcome, end of warfarin exposure or death. Simple linear interpolation [14] was applied to classify each day of follow-up in predefined therapeutic range categories (INR <2, INR 2–3, INR >3, INR unknown). For each patient, periods of INR values of in-range (2–3) and out-of-range (<2 and >3) were recorded. The period without INR test results was classified as “unknown”. Clinical outcomes including the first inpatient occurrence of thromboembolic events (ACS, ischemic stroke, TIA, systemic embolism) and major bleeding were observed during the same follow-up period.

### Data Analyses

Baseline characteristics including demographic and clinical characteristics were summarized with descriptive analyses. The number of patients with clinical outcomes and event rates per 100 patient-years of each INR range were calculated.

The relationship between clinical outcomes and out-of-range INR values was estimated using the multivariate Poisson regression model to control for differences in patient age, gender, region, race, Charlson Comorbidity Index (CCI) score, baseline treatment and individual comorbidities. Poisson regression was applied to adjust for repeated measures and is more appropriate for INR measures since it is a count model. Relative risk ratios (RRRs) and 95 % confidence intervals (CIs) are reported, by comparing the risk during the time periods when INR was in-range versus when INR was out-of-range. An additional analysis was performed replicating the clinical outcome Poisson regression during the first 6 months and after 6 months of warfarin initiation.

## Results

The study population included 34,346 patients diagnosed with NVAF who initiated warfarin prophylaxis. Table 1 shows the baseline characteristics of these patients. The mean age of NVAF patients receiving initial warfarin prescriptions was 71 years, and 98.36 % were male. Most patients were White (69.53 %) and resided in the South US region (34.61 %). Hypertension (75.87 %) was the most common comorbidity followed by diabetes mellitus (39.53 %) and vascular disease (39.02 %). Based on CHADS_2_ risk scoring [15, 16], 89.34 % patients were at moderate or high-risk of stroke.

**Table 1:** Baseline demographic and clinical characteristics of warfarin patients with NVAF

	Warfarin patients with NVAF (N = 34,346)	
	N/Mean	%/SD
Age		
Age (Mean)	70.98	10.22
Age 18–64	10,938	31.85 %
Age 65–74	9,608	27.97 %
Age 75+	13,800	40.18 %
Gender		
Male	33,783	98.36 %
Female	563	1.64 %
US geographic location		
Northeast	4,988	14.52 %
Midwest	8,612	25.07 %
South	11,914	34.69 %
West	7,078	20.61 %
Other	1,754	5.11 %
Race		
Black	2,673	7.78 %
Hispanic	1,092	3.18 %
White	23,882	69.53 %
Other	6,699	19.50 %
Baseline comorbid condition		
Indices		
Charlson comorbidity index score	1.98	2.03
Chronic disease score	7.68	4.50
CHADS_2_ score		
Low (0)	3,662	10.66 %
Moderate (1)	9,154	26.65 %
High (>1)	21,530	62.69 %
CHADS_2_-VASc Score		
Low (0)	1,976	5.75 %
Moderate (1)	4,192	12.21 %
High (>1)	28,178	82.04 %
ATRIA score		
Low (0–3)	27,279	79.42 %
Moderate (4)	2,451	7.14 %
High (5–10)	4,616	13.44 %
Comorbidities		
Congestive heart failure	6,962	20.27 %
Diabetes mellitus	13,577	39.53 %
Hypertension	26,060	75.87 %
Ischemic stroke	1,652	4.81 %
Transient ischemic attack	819	2.38 %
Vascular disease	13,403	39.02 %
End-stage renal disease	391	1.14 %
Hospitalized hemorrhagic stroke	1	0.00 %
Chronic kidney disease	4,418	12.86 %
Chronic obstructive pulmonary disease	1,667	4.85 %
Anemia	4,714	13.73 %
Hospitalized major bleeding	153	0.45 %
Bleeding disorder	572	1.67 %
Baseline medications		
Rate control medication use	22,430	65.31 %
Rhythm control medication use	2,612	7.60 %
Aspirin use	10,351	30.14 %

NVAF, non-valvular atrial fibrillation; SD, standard deviation; CHADS_2_ score, congestive heart failure (point = 1); Hypertension (point = 1), Age ≥ 75 years (point = 1), Diabetes mellitus (point = 1), prior Stroke or transient ischemic attack (point = 2); CHADS_2-_VASc score^16^, congestive heart failure (point = 1); Hypertension (point = 1), Age ≥ 75 years (point = 2), Diabetes mellitus (point = 1), prior Stroke or transient ischemic attack (point = 2); Vascular disease (point = 1), Age 65–74 years(point = 1), Female category (point = 1); ATRIA, anticoagulation and risk factors in atrial fibrillation bleeding risk score (factors: anemia, severe renal disease, age ≥ 75 years, previous hemorrhage, diagnosed hypertension)

There were 1,372 ACS events, 1,045 ischemic strokes, 297 TIA events, 189 systemic emboli and 726 major bleeding events that required hospitalization observed during the warfarin exposure period. Table 2 provides incidence rates per 100 person-years for each of the clinical outcomes. All event rates were highest when patients had INR <2, even for major bleeding.

**Table 2:** Incidence rates by INR range

	Incidence per 100 patient-years		
	INR <2	INR 2–3	INR >3
Ischemic stroke	10.3	1.3	0.72
TIA	2.9	0.33	0.23
Systemic embolism	1.8	0.26	0.12
ACS	13.7	1.6	1.5
Major bleeding	4.6	1.6	2.6

*INR* international normalized ratio, *TIA* transient ischemic attack, *ACS* acute coronary syndrome

**Fig. 1:**
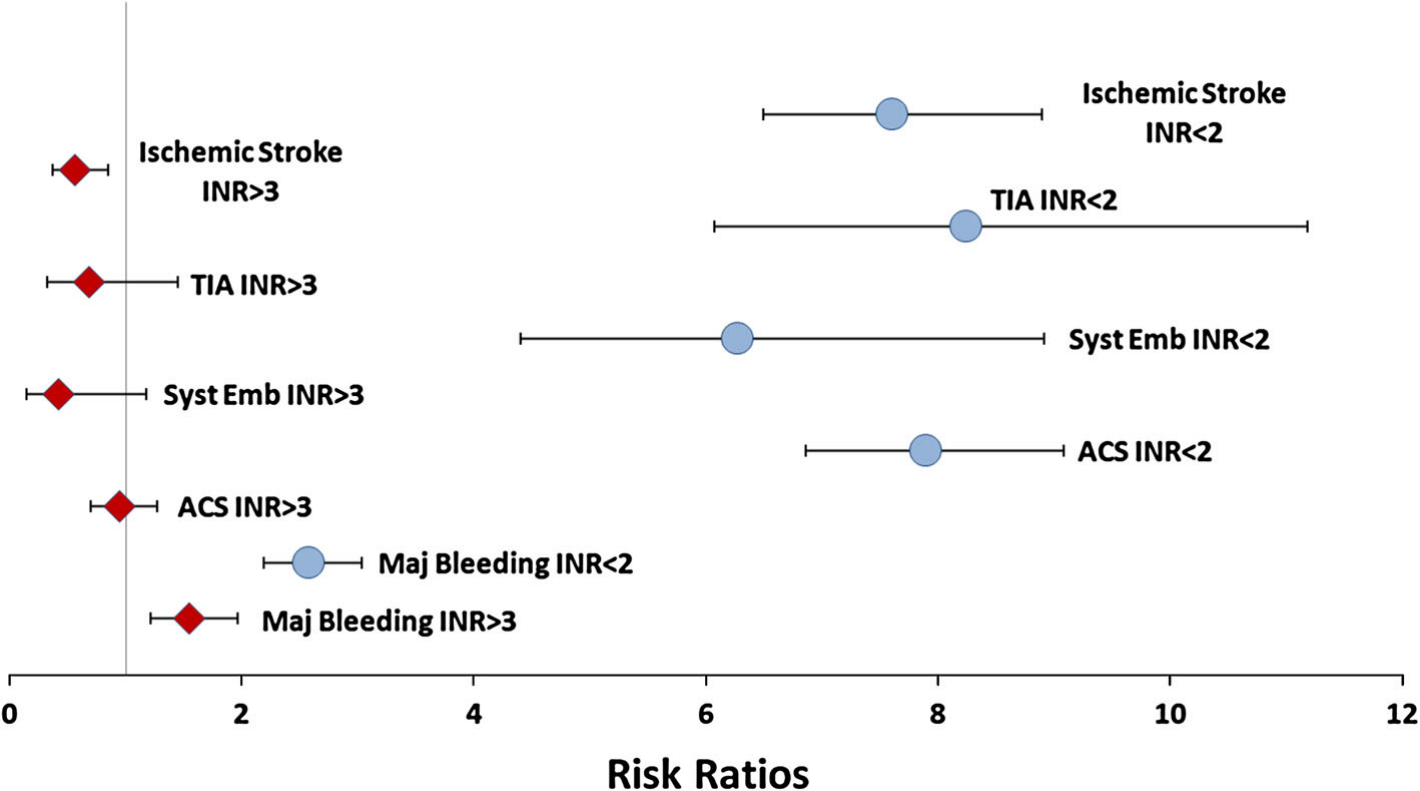
Adjusted RRRs for clinical outcomes, out-of-range versus in-range. *RRR* relative risk ratio, *INR* international normalized ratio, *TIA* transient ischemic attack, *ACS* acute coronary syndrome; *Syst Emb* systematic embolism. *Note*: A relative risk ratio of higher than 1 indicates a higher likelihood of having the clinical outcomes as compared to in-range international normalized ratio time periods. The *horizontal bars* for each relative risk ratio correspond to the 95 % confidence intervals. If the 95 % confidence interval exclude 1, the relative risk ratio estimate is statistically significant

The association between clinical outcomes and out-of-range INR is presented in Fig. 1, including respective relative risk estimates and CIs. After controlling for patient characteristics, during time periods with INR <2 exposure, patients had significantly increased risk of developing ACS (RRR = 7.89, 95 % CI = 6.85–9.08) compared to those with INR values between 2 and 3. Similarly, during time periods with INR <2, patients were found to be associated with significantly increased risk of developing ischemic stroke (RRR = 7.60, 95 % CI = 6.50–8.89), TIA (RRR = 8.24, 95 % CI = 6.08–11.18), systemic embolism (RRR = 6.27, 95 % CI = 4.40–8.92) and major bleeding (RRR = 2.58, 95 % CI = 2.19–3.03), compared to periods with INR of 2–3. During time periods with INR >3, patients were found to be associated with significantly higher risk of major bleeding (RRR = 1.55, 95 % CI = 1.21–1.97) than those with INR of 2–3. During time periods with INR >3, patients were associated with significantly decreased risk (RRR = 0.56, 95 % CI = 0.37–0.85) of developing ischemic stroke compared to those with INR of 2–3.

To examine the association between out-of-range INR and adverse clinical outcomes over time, Poisson regression was performed to compare the periods before and after the first 6 months of warfarin exposure (Table 3). For the period of exposure to INR <2, the significantly increased risks for adverse clinical events were consistent before and after 6 months of warfarin use for all clinical outcomes, except for TIA. For TIA, the RRR diminished after 6 months of warfarin use, and was not statistically significant. For thromboembolic events, the magnitudes of the RRRs were higher during the first 6 months compared to that after 6 months. Conversely, for major bleeding, the RRR was higher after 6 months. For the period of exposure to INR >3, the difference was less obvious between the periods before and after 6 months. There was a slight increase in the risk of major bleeding during the first 6 months of warfarin use; this increased risk remained after 6 months, although it was no longer statistically significant.

**Table 3:** Relative risk estimates for clinical outcomes during and after the first 6 months of warfarin exposure

Clinical outcomes				
INR	Before 6 months		After 6 months	
	Relative risk	*p-*value	Relative risk	*p*-value
ACS				
<2	6.6505	<0.0001	4.3918	<0.0001
>3	0.8847	0.4645	0.7998	0.5357
Ischemic stroke				
<2	6.8189	<0.0001	2.9368	<0.0001
>3	0.5362	0.0090	0.4596	0.0967
Transient ischemic attack				
<2	8.9904	<0.0001	1.8577	0.0768
>3	0.8693	0.7378	0.2505	0.1769
Systemic embolism				
<2	4.8545	<0.0001	4.5573	0.0067
>3	0.2138	0.0338	1.8882	0.4476
Major bleeding				
<2	2.0007	<0.0001	3.0921	<0.0001
>3	1.4562	0.0120	1.5150	0.0555

INR 2–3 = Reference group

*ACS* acute coronary syndrome, *INR* international normalized ratio

## Discussion

Using national VHA data, this study assessed the relationship between out-of-range INR values and clinical outcomes among 34,346 NVAF patients who were newly prescribed warfarin prophylaxis. The risks of thromboembolic events were 6.3–8.2 times higher during periods when patients had INR <2, compared to periods with an INR of 2–3. Conversely, risk of a bleeding event was 1.5–2.6 times higher when INR was outside of the 2–3 range, as compared to when INR was within that range. The study confirmed previous results [17], while demonstrating the particularly high magnitude of risks associated with below-range INRs. Thromboembolic risk was also heightened during the first 6 months of warfarin therapy when patients were exposed to below-range INR values.

The current study results were consistent with a number of studies that have shown an association between supra-therapeutic INR (INR >3) and increased bleeding rates among patients prescribed warfarin. Hylek et al. [18] found a nine times higher odds of intracranial bleeding among patients with INR higher than 4.5 at the time of a stroke. Sarawate et al. [19] found a 1.72 higher adjusted odds of major hemorrhage among AF patients who had an INR of >3 upon hospital admission. Tapson et al. [20] observed that 9 % of patients with or at risk for thromboembolic diseases had INR >4 during a hospitalization, and 0.8 % of patient experienced major hemorrhage, although this study did not correlate high INR with bleeding events. In addition, the current study showed the increased risk of bleeding during outpatient exposure to supra-therapeutic INR.

Conversely, sub-therapeutic INR (INR <2) increases the risk of developing thromboembolic complications. In a large cohort study of 13,559 NVAF patients, those with INR values <2.0 showed an increased risk of stroke compared with those whose INR was within the therapeutic range (*p* = 0.03) [18]. A systematic review of 47 studies showed that a 12 % improvement in target therapeutic range prevents one thromboembolic event per 100 patient-years [21]. The current study adds to the previous literature by quantifying the magnitude of the increased risks when patients were exposed to below-range INR.

Surprisingly, in this population, sub-therapeutic INR was associated with bleeding events. This may indicate downward adjustments of warfarin dosing in response to the bleeding event. Because the Rosendaal method interpolates INR levels between two INR values, the analysis could not determine the actual INR value on the bleeding event date. This limitation of the Rosendaal method means that the RRR estimates of the current analysis may be an underestimation, as clinicians may have made corrective adjustments to the warfarin dose in response to thromboembolic or bleeding events. The Rosendaal method is the proven standard method for this type of analysis. To our knowledge, prior research studies have not compared bleeding risk among patients with warfarin INR <2 to those with in-range INR. A recently published meta-analysis conducted by Mearns et al. [22] showed that only 42 % of hemorrhagic events occurred among patients exposed to warfarin INR at >3.0, indicating that more than half of the bleeding events occurred when INR was <3.0. Our findings provide the hypothesis: when INR is <2.0, the bleeding risk may be higher than when INR is within the 2.0–3.0 range. Future research is warranted to investigate and confirm our findings.

The current study was subject to several limitations. While claims data are valuable for the examination of health outcomes, treatment patterns, and costs, claims data are collected for the purpose of payment and not research. The presence of a claim for a filled prescription does not indicate that the medication was consumed or that it was taken as prescribed. In the current analysis, the presence of INR testing and refill data of warfarin increased the certainty that warfarin was indeed consumed. In addition, diagnostic codes in claims data may contain inaccuracies or omissions. These inaccuracies are expected to occur randomly, however, and are unlikely to have significantly impacted the findings. Finally, a general limitation of claims data analysis is that only observable factors were used, and there may be residual confounding due to unmeasured clinical and disease-specific parameters. Despite these limitations, the large population and extended follow-up allowed the observation of clinical impact of exposure to sub-optimal INR level.

The results obtained using the VHA claims database may not be generalizable to other populations with NVAF. Importantly, the VHA population contains very few women, and veterans may have additional or different risk factors as compared to the general population. The conclusions drawn from this study should be cautiously interpreted, and additional data from other populations is needed.

## Conclusion

In this large VHA population, NVAF patients newly prescribed warfarin for stroke prophylaxis had significantly higher risk of adverse clinical outcomes, including ACS and TIA, during periods of exposure to out-of-range INR values. The risks of thromboembolic events associated with below-range INR were especially high, indicating substantial danger to patients when warfarin prophylaxis doses are subtherapeutic.
